# Factors used by general practitioners for referring patients with chronic musculoskeletal pain: a qualitative study

**DOI:** 10.1186/s12875-022-01743-6

**Published:** 2022-05-24

**Authors:** Syl Slatman, Annemiek Mossink, Duncan Jansen, José Broeks, Peter van der Lugt, Gert-Jan Prosman, Wendy Oude Nijeweme - d’Hollosy

**Affiliations:** 1 Department of Psychology Health & Technology, University of Twente, Faculty of Behavioral, Management & Social Sciences (BMS), De Zul 10, 7522NJ Enschede, the Netherlands; 2 Department of EEMCS/BSS, University of Twente, Enschede, the Netherlands; 3 Department of Rehabilitation, Zorggroep Twente, Almelo, the Netherlands; 4 Roessingh Rehabilitation Center (Department of Chronic Pain), Enschede, the Netherlands; 5 Research Department General Practice, General Practitioner Cooperative Twente (THOON), Hengelo, the Netherlands

**Keywords:** Chronic musculoskeletal pain, Referral factors, General practitioners, Semi-structured interviews

## Abstract

Around 20% of the Dutch population is living with chronic musculoskeletal pain (CMP), which is a complex and multifactorial problem. This complexity makes it hard to define a classification system, which results in non-satisfactory referring from the general practitioner (GP). CMP is often explained using the biopsychosocial model in which biological, psychological and social factors cause and maintain the pain. The presented study investigated the factors related to the GPs’ referral for patients with CMP to further treatment.

Using convenience sampling, semi-structured interviews and a focus group were conducted among 14 GPs. The interviews were iteratively analyzed using inductive conventional content analysis.

Analysis of the interviews demonstrated that there were 28 referral factors that were mentioned by more than 50% of the interviewed GPs. The results showed that the GPs were mostly focussing on the physical (e.g. pain location) and psychological (e.g. acceptation of pain) factors, indicating that they lack focus on the social factors. Furthermore, unfamiliarity of GPs with treatment options was a noteworthy finding.

The referral of patients with CMP by GPs is complex and based on multiple factors. To improve referral, it is recommended to include social factors in the decision-making process and to increase the familiarity of the GPs with available treatments.

**Supplementary Information:**

The online version contains supplementary material available at 10.1186/s12875-022-01743-6.

## Background

Around 20% of the Dutch population is living with from chronic musculoskeletal pain (CMP) [1, 2]. CMP is defined as pain lasting longer than 3 months. Patients with CMP report a lower quality of life and CMP is associated with problems like difficulties with activities of daily living (ADLs), depression and other mental health problems [2]. CMP is a complex problem due to the interplay of biological, psychological and social factors on the development and persistence of CMP [3, 4].

The complexity of CMP and the frequent presence of comorbidity with psychological complaints complicates the development of one single valid classification system to categorize patients with CMP, which leads to insufficient quality of referring [5]. This results in a diminished quality of care, due to healthcare providers sending back patients to their general practitioner (GP) when treatment is not effective and leads to higher healthcare costs [6]. A previous study found that more than 30% of the GPs’ referrals were potentially avoidable [6]. Common healthcare providers that treat chronic pain include physiotherapists, occupational therapists, medical specialists and mental healthcare professionals [5].

In the Netherlands, the GP is usually central in the patients’ treatment as the gatekeeper within the Dutch healthcare system [7]. So, to improve the quality of referring, it is crucial to better understand the factors GPs use for referring patients with CMP. Itz, Huygen and van Kleef [8] point out the importance of GPs evaluating the risk factors for chronicity and explaining the treatment plan to the patient. This might help in selecting the appropriate treatment for patients with CMP earlier. Additionally, Pitt, O’Conner and Green [9] pointed out that the GPs’ familiarity with different treatment options for osteoarthritis is an important factor for referring. For example, their knowledge about self-management programmes was insufficient, which influenced their referral. Most studies focused on a specific target group such as low back pain [8] or osteoarthritis [9], but little research has been done with a focus on referring patients with CMP in general.

To improve the referral of patients with CMP it is crucial to first get more insight in factors GPs focus on when referring. To accomplish this, the aim of this study was to identify those factors used by GPs when referring patients with CMP for further treatment.

## Method

### Research design and participants

This research is part of a larger project called PReferral, that is focused on the design of a decision support tool to support the GPs in the referral of patients with CMP.

This explorative qualitative study, analysed using conventional content analysis, took place in the east of the Netherlands (Twente) among practicing GPs. A qualitative design was chosen, because referral of patients (with CMP) to treatment is a complex process [10] of which theoretical background is lacking. In the first phase, 10 semi-structured interviews with GPs were conducted about factors related to the referral of their patients with primary or secondary chronic pain. In the second phase, the results of these interviews were verified and supplemented where necessary by a focus group with 4 GPs. For this second phase, a focus group was chosen because this enabled social interaction, which could yield referral factors that were not identified in the interviews [11]. Using convenience sampling 139 GPs were approached of which 14 GPs participated in this research, a response rate of 10%. The demographics of the interviewed and focus group GPs are respectively presented in Tables 1 and 2. The study was approved by the ethics committee of the University of Twente (approval number: 201287).

**Table 1:** Demographics of interviewed GPs

Characteristic		n	Median (range)
Sex	Male	5	
	Female	5	
Working in	City	3	
	Village	7	
Kind of practice	Group-practice	7	
	Solo-practice	3	
Age (years)			50 (34–63)
Experience as GP (years)			15.5 (2.5–31)
Professional interest in CMP (0–10)			6.5 (5–7.5)
Satisfaction with referring CMP patients (0–10)			5.5 (2–7.5)

**Table 2:** Demographics of focus group GPs

ID	1	2	3	4
Sex	F	M	M	M
Working in	Village	Village	City	Village
Kind of practice	Group	Solo	Group	Group
Age (years)	60	43	50	55
Experience as GP (years)	26	10	16	25
Professional interest in CMP (0–10)	8	3	7	7
Satisfaction with referring CMP patients (0–10)	5	7	6	7

### Measures/materials

The Dutch version of the semi-structured interview scheme (Additional file 1) consisted of 33 open- and closed-ended questions which were based on the literature and consensus of the researchers (S.S., A.M., J.B., G.P.). Using the open questions, the participants were encouraged to give examples during the interview. The focus group used the results from the interviews to verify and supplement (Additional file 3).

### Procedure

The GPs were contacted for the interviews using a newsletter of two GP organizations, named THOON and FEA, with a link to additional information about the study. Furthermore, a list of GPs was made by these organisations and GPs were approached personally by phone by a student from the University of Twente (A.M.). Moreover, a rehabilitation doctor of a local hospital (ZGT) (J.B.) contacted GPs that often referred to the ZGT. The GPs for the focus group were contacted by the research coordinator of THOON (P.L.), using convenience sampling. After agreeing to be interviewed or participate in the focus group, the participants answered demographic questions (i.e. sex, location of practice, type of practice, age, and years of experience as GP), a question about their professional interest in patients with CMP on a scale from 0 to 10 and their satisfaction with their referral of patients with CMP on a scale from 0 to 10. The interviews were conducted by one researcher (A.M.) and the focus group was conducted by one researcher (S.S.) and moderated by another researcher (J.B.). Because of COVID-19, the conducted interviews and focus group were online via Microsoft Teams and the interviews lasted between 25 and 84 minutes, with a median of 54 minutes and the focus group lasted 79 minutes. All participants were informed about the project goals beforehand and gave verbal informed consent to participate in the study and be recorded. The interviews and focus group were recorded in Microsoft Teams, transcribed using Amberscript software and manually corrected by the researchers. The recordings and transcripts of the interviews and focus group are stored in a secured online environment of the University of Twente.

### Data Analysis

The data of the interviews was analysed in Atlas.ti, using inductive conventional content analysis [12]. This method was deemed most appropriate, because existing literature about GPs’ referral factors for patients with CMP is limited [13]. The first step in the iterative process was for the two researchers (S.S. and A.M.) to separately read all transcripts freely. In the second step, these two researchers independently generated initial codes using meaningful words and sentences of three interview transcripts, using the following question: “which factors do GPs use for the referral of patients with CMP?”. Subsequently, these codes were discussed and fine-tuned with three researchers (S.S., A.M., D.J.) until consensus was reached. This was repeated iteratively with the remainder of the interviews, until all data was analysed. Next, the identified codes were categorized and developed in themes by three researchers (S.S., A.M., D.J.). A priori it was decided to use a benchmark of 50%, meaning that at least half of the GPs had to mention a code in order to describe it in the results. This was done because a large amount of codes was expected to be mentioned, of which we wanted to extract the most important ones. To analyse the interrater reliability, Cohen’s Kappa was calculated based on the coding of one random interview by two researchers (S.S., A.M.). The level of agreement was categorized according to the Kappa values as none (0–.20), minimal (.21–.39), weak (.40–.59), moderate (.60–.79), strong (.80–.90) and almost perfect (>.90) [14]. For this interview, Cohen’s Kappa was 0.67, which means that the agreement between the two researchers was moderate. Finally, the results were discussed with all authors and consensus was reached on both the themes and factors GPs used for referral of patients with CMP. The input of the focus group was checked for both known and new codes. This was done independently by two researchers (S.S. and J.B.), using Microsoft Word and discussed with all authors until consensus was reached.

## Results

**Fig. 1:**
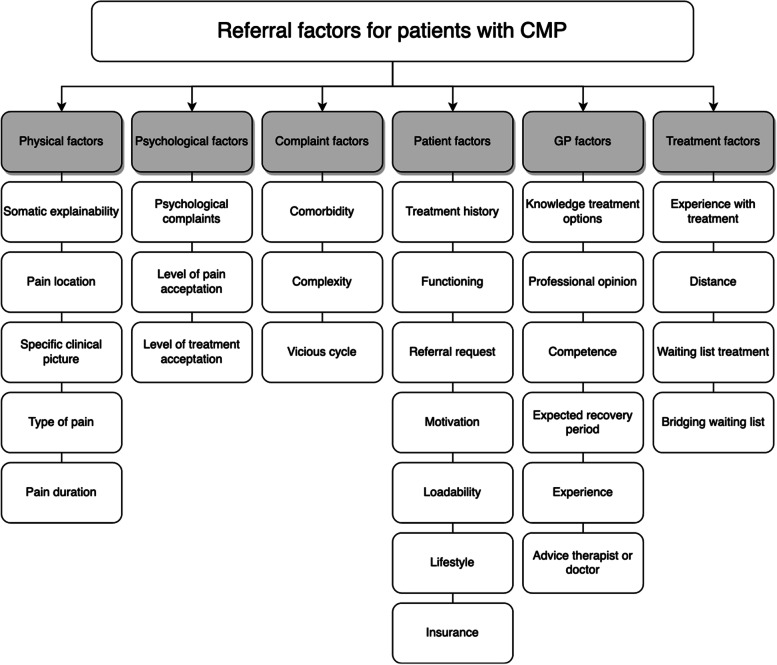
Final coding scheme with themes and codes

In total, 83 factors for referring patients with CMP were stated by the interviewed GPs (Additional file 2). These 28 factors (34% of total factors) were divided in six themes and are explained per theme. The factors that were mentioned by 50% or more of the interviewed GPs, are presented in Fig. 1.

### Physical factors

The physical factors, as shown in Table 3, were related to the somatic aspects of CMP, for example this could have been about the location or duration of the pain.

**Table 3:** Explanation of the factors within the theme “physical factor”

Factors	Definition	Quote
Somatic explainability	The physical explainability of the complaint, more specific when there was a known underlying physical cause that explains the pain of the patient.	*“If you are still on an organic substrate, you are often looking into neurology and orthopaedics.”* (GP 6)
Pain location	The pain of the patient could be located in different body parts such as the neck, knee, hip or back.	*“If you have more or less solitary back or neck complaints, say for spine-related complaints …*” (GP3)
Specific clinical picture	There was a diagnosis for the complaints of the patients which influenced the referral.	*“I refer people with herpes or shingles, they have pain complaints for possibly several options.”* (GP8)
Type of pain	A specific type of pain, for example neurological pain, oncological pain or posture-related pain	*“...I refer to them when I think it mainly concerns neurogenic pain.”* (GP2)
Pain duration	This code referred to the period a patient was suffering from the pain before they were referred.	*“...quite in the beginning, so if someone quite recently got back pain, they are going to the physiotherapist pretty quickly.”* (GP7)

### Psychological factors

The psychological factors, explained in Table 4, contained the cognitive or emotional aspects of CMP, these factors were about the mental state of the patient.

**Table 4:** Explanation of the factors within the theme “psychological factors”

Factors	Definition	Quote
Psychological complaints	Different psychological complaints patients suffered from, including feelings of depression, anxiety or trauma related mental health problems.	*“...if there really is underlying, real psychological suffering, so, if there is also depression and anxiety, especially much more in the foreground.”* (GP1).
Level of pain acceptation	The level of acceptation of the patient, including the level of acceptation that pain was chronic and the level of acceptation that there was no physical cause to explain the pain.	*“With patients who are open for and realise their pain is chronic and there is not a physical cause and they have to live with it, you can often go to primary care.”* (GP2).
Level of treatment acceptation	The acceptation of the treatment offered by the GP. The patient should be open for trying out the treatment and treating the psychological complaints instead of the physical complaints.	*“There is no point in referring people to the SOLK-clinic if they do not support it. That is pointless, completely pointless.”* (GP5).

### Complaint factors

The complaint factors, as shown in Table 5, were about the pain and possible other problems occurring within the patient which were contributing to the complaint of the patient with CMP.

**Table 5:** Explanation of the factors within the theme “complaint factors”

Factors	Definition	Quote
Comorbidity	The occurrence of two or more complaints in one patient at the same time.	*“Especially if there are indeed often several problems in play…. for example. I have had a patient with a car accident and he does indeed continue to have chronic neck pain, but also has concentration problems and forgetfulness, so several complaints in several areas”* (GP1).
Complexity	The complexity of the pain, estimated by the GP as more or less complex.	*“...for more complex problems, I refer to Roessingh (3rd line care) and for simpler problems to Medinello (2nd line care)”* (GP3).
Vicious cycle	Patients who were experiencing struggles in multiple domains, often psychological or physical problems combined with social or work problems. These different types of problems increased the other problem, which caused a circularity which was hard to break.	*“When a patient gets stuck in a vicious cycle, for example: the patient does not move or exercise anymore because of the pain.”* (GP5).

### Patient factors

The patient factors, discussed in Table 6, contained the factors associated specifically to the person suffering from the CMP and the daily life of the patient.

**Table 6:** Explanation of the factors within the theme “patient factors”

Factors	Definition	Quote
Treatment history	Patients with CMP often had treatment before. Earlier referrals could be for diagnostics, physical treatment, psychological treatment or medicine use.	*“Before you refer there, most of the patients are patients with whom you have had, of course, a part of the trajectory where you have already had physical therapy, have used several things…”* (GP1).
Functioning	Daily functioning, such as doing the household, and physical functioning, such as being able to walk.	*“...if there really is a specific act or function in which they are in a lot more pain, then an occupational therapist is very helpful.”* (GP5).
Referral request	The patient could have a request for a referral to a specific treatment or institution but this might also be a request for a somatic treatment instead of a psychological consult.	*“…and in 90% of the back clinic, it is mainly the back pain patients who go that way with a request.”* (GP1)
Motivation	Motivation drove a patient to a certain behaviour and commitment to the treatment. This code was used when it was not just about being open for a treatment but also being committed and willing to invest.	*“...some patients say: yes, I want to do anything about it.”* (GP9).
Loadability	The enduring capacity that an individual has both on the mental and physical level.	*“Looking at most chronic patients I have or the fibromyalgia patients, they are more likely to go to a psychosomatic physiotherapist or a psychologist, because they often have load-bearing problems too.”* (GP3).
Lifestyle	The patient had a way of living with certain habits that have influenced their health, for example smoking or having overweight.	*“This was in regard to an overweight woman...”* (GP10).
Insurance	In the Netherlands, people get to choose their own insurance company and package and not every company or package covered every treatment.	*“...with physiotherapy, patients are quickly at the point where they have to pay for their treatment, which certainly plays a role.*” (GP2).

### GP factors

The GP factors were associated with the different aspects related to the GP, as the referrer of the patient with CMP. These factors are discussed in more detail in Table 7.

**Table 7:** Explanation of the factors within the theme “GP factors”

Factors	Definition	Quote
Knowledge treatment options	The GP was not always familiar with the different options for referring, the possibilities within a treatment or which patients were accepted in a certain clinic.	*“Okay, the psychosomatic therapist and psychomotor therapist, I thought it was kind of the same.”* (GP7).
Professional opinion	The opinion of a GP about the treatment or the healthcare provider of a clinic.	*“No, you don’t have that many exercise therapists in our area who do that. We had one in the village, but I didn’t think he was good.”* (GP7).
Competence	The ability to understand the patient and the complaint were influencing the referral. The competence of the GP to diagnose or treat the patient influenced the referral.	*“Yes, looking at fibromyalgia, I can officially make that diagnosis myself according to the guidelines. But it is such a loaded diagnosis that I often choose to refer them anyway, so that I actually do a second opinion of myself.”* (GP3).
Expected recovery period	When a patient was presenting their complaints to the GP, an estimation was made of the time the complaint would precede.	*“...if it is something for a shorter project, if I estimate we can tackle the complaint a bit faster and in a more active way.”* (GP4).
Experience	The experience of the GP with the patient and the complaint. The GP did refer to his experience in the working field as a criterium for referring patients.	*“…based on my experience, intuition and offer.”* (GP6).
Advice therapist or doctor	The GP or patient could have received an advice for a referral from a specialist, therapist or company doctor. This advice did not always result in a referral	*“Sometimes people come up with the story that they need a referral from the company doctor for an MRI or something.”* (GP2).

### Treatment factors

The treatment factors, shown in Table 8, included the factors related to the medical treatment options, according to the GPs.

**Table 8:** Explanation of the factors within the theme “treatments factors”

Factors	Definition	Quote
Experience with treatment	The experiences of the GP and the patient with a certain practitioner that influenced the referral.	*“In the first line, of course, we have the regular physiotherapist and the psychosomatic therapist, but I must say that I mainly refer to the Cesar therapist because we have good experiences with them”* (GP1).
Distance	The distance between the hometown of the patient and the healthcare provider.	*“Patients in Haaksbergen think it is quite a big deal to go to Enschede for a physiotherapist or an occupational therapist”* (GP6).
Waiting list treatment	Healthcare institutions in the region Twente had varying waiting times and GPs took these into consideration when referring.	*“…and then I prefer to refer a patient to the rehabilitation clinic, because the waiting time is not too bad”* (GP5).
Bridging waiting list	Sometimes patients were on a waiting list for treatment and were referred to another healthcare provider to bridge the time on the waiting list.	*“Yes, then I try to use the practice nurse mental health to bridge the gap.”* (GP1).

### Focus group

The focus group received aforementioned results and verified found themes and factors by explicitly confirming these results and mentioning referral factors from each of the six identified categories. Moreover, some additional factors for referring patients with CMP were mentioned in the focus group. These additional factors were also mentioned by interviewed GPs, but not by more than 50% of the GPs, as shown in Table 9.

**Table 9:** Results focus group

Factors	Theme	Mentioned by interviewed GPs (n)
Availability treatment	Treatment factors	3
Singular complaint	Complaint factors	2
Specific request for help	Patient factors	4

## Discussion

The present study aimed to investigate the factors related to GP referral of patients with CMP for further treatment. In total, 83 factors were found that influenced the referral of patients with CMP, of which 28 factors were mentioned by 50% or more of the interviewed GPs and verified by the focus group. All interviewed GPs within this study mentioned the somatic explainability, location of the pain complaints, psychological complaints, the treatment history, physical functioning of the patient and referral request of the patient as specific factors influencing the referral of patients with CMP. The found factors in this study were categorized in the following six themes: GP, treatment and patient (physical, psychological, complaint, general) factors. A seventh theme was identified, called “external factors”, with factors like social environment and financial situation. However, none of the factors in this theme were mentioned by 50% or more of the GPs and therefore this theme was not discussed in the results. The six identified categories, correspond to previous studies that found that the GPs’ referral is based on (1) GP factors, (2) treatment factors and (3) patient factors [10, 11].

According to the guidelines of the Dutch GP organization, a combination of physical, psychological and social factors is contributing to and causing CMP [13, 15]. Additionally, many of the referral factors found in this study are mentioned in this guideline, like risk factors for chronicity (e.g. pain duration, comorbidity and psychological complaints) and diagnostic factors (e.g. location of pain, somatic explainability and functioning). The guidelines mention social factors as one of the main contributing and causal explanations of chronic pain, however in this study not a single social factor was mentioned as a referral factor by at least 50% of the GPs. This is noteworthy, since the biopsychosocial approach is crucial for the understanding and treatment of CMP [16]. Social factors that are related to CMP include social support [17, 18], social isolation, [19, 20] and job satisfaction [21, 22]. Our findings are in line with prior studies on referral factors, that found that biomedical elements and GP factors are most important in the referral process [23, 24]. The GPs’ identification of the social environment of their patients is limited and they are having difficulties estimating the loneliness and social participation of their patients, despite them being aware of the consequences for their health and health perception [25, 26]. Hansen, Rosendal, Fink and Risor [27] focused on patients with medically unexplained symptoms and found that GPs seldom act on psychosocial cues. One possible explanation for this could be that due to time constraints, GPs mainly focus on identifiable and treatable pathology [28] and disregard social factors, as described in our results. The biopsychosocial model is supported with empirical evidence, but in practice the psychosocial factors are often viewed as secondary and as a reaction to the pain [29]. Moreover, Knoop et al. [30] found that guidelines for chronic low back pain vary widely regarding recommendations for prognostic psychosocial factors. These studies might explain why GPs do not focus on the social factors when referring patients with CMP. When the GP is not familiar with the social environment of the patients, it will not be used as a factor for referring which might lead to a suboptimal referral. Furthermore, these studies confirm that the referral of patients with CMP is very complex [28]. This can also be concluded based on the finding that a total of 83 referral factors were found in this study, but only 34% of these factors were mentioned by 50% or more of the GPs and the complexity of referring was confirmed and again explicitly mentioned by GPs in the focus group.

Within this study, 90% of the interviewed GPs mentioned their unfamiliarity with treatment options as an influencing factor for their referrals of patients with CMP. This unfamiliarity was either about unfamiliarity with the content of a certain treatment or the presence of this treatment in the region. In relation to the physiotherapy related treatments, it was mentioned GPs often did not seem to make a distinction between the different forms of physiotherapy, because of unfamiliarity with these therapies. For example, it is hard for GPs to make a distinction between manual physiotherapy and regular physiotherapy, resulting in referring towards the more familiar option, regular physiotherapy [31]. Previous studies found that the unfamiliarity with treatment options could be a barrier for referring towards non-pharmacological treatment [32, 33], like self-management programmes [12]. The unfamiliarity with treatment options is in contrast with the referral factor “availability of treatment options”, which was mentioned by 40% of the interviewed GPs and in the focus group. This suggests that there might be a blind spot for certain treatment options for patients with CMP, possibly accounting for incorrect referrals.

### Strengths and limitations

This study was the first study to identify factors GPs use to refer patients with CMP. Another strength of this study was the sample, with a wide range in age, years of experience as a GP and satisfaction with referring, which increased the representativeness of the factors found within this study. Furthermore, the study design had two phases, where the results of the interviews were verified by the focus group, which increased thoroughness. Also, the interpretation and coding of the interviews by multiple researchers was a strength. Constantly discussing and checking the codes with multiple researchers ensured an open-minded approach for creating codes.

The willingness of GPs to cooperate to this research was an important limiting factor in this study. The GPs indicated that their time and focus was on the COVID-19 pandemic, because a lot of changes had to be made within primary care [34]. A large number of GPs were approached via an online article but only a few participated. This might have caused a selection bias and the sample might not be representative for (the east of) the Netherlands. Additionally, the sample size might have been too small, given that additional referral factors were found in the focus group. However, it should be noted that these referral factors were mentioned by some of the GPs, but did not meet the 50% benchmark. Furthermore, the focus group was not analysed following all steps of qualitative research, which possibly could have let to missed referral factors. Also, due to the subjective nature of qualitative research, some factors and themes overlap and might not be as distinctive as reported in this study.

### Suggestions for further research

This study specifically focused on the referral of patients with CMP by the GPs in the east of the Netherlands. Further research should expand the region and number of the participants to increase the representability. By interviewing more GPs, either using interviews or focus groups, it will be possible to allocate more detailed weight to referral factors and to gain more insight in referrals to different kind of treatments. Specifically, the reason why social factors currently seem to be overlooked in the referral of patients with CMP should be further investigated. Also, this study provides factors GPs use for the referral of patients with CMP, but does not give an explanation as to why these factors are used for referral. Additionally, this study covered patients with both primary and secondary chronic pain, while it might be possible that there are different referral factors for these groups of patients. Further research could specify a patient group to get better insight in referral factors for specific chronic pain conditions.

### Practical implications

Based on the results of this research, it is evident that GPs should take social factors into account when referring patients with CMP. This could be supported by promoting the use of guideline suggested tools, like the SCEGS (somatic, cognitive, emotional, behaviour and social) method [35].

Further, unfamiliarity of the GP with the different treatment options seems to be an important factor for their referral. An implication for improving the familiarity of the GPs, would be to support them in their awareness of treatment options for patients with CMP. For example, by developing an easily accessible and usable (digital) tool or eHealth application, in which all treatment options and characteristics in this region are mentioned, including both mono- and multidisciplinary treatment options. On the other hand, there is a public social map in the Netherlands where patients are able to see possible treatments near to them. Therefore, the responsibility of finding the most fitting treatment could also be shared between the GP and the patient.

## Conclusion

Concluding, this study identified different factors important for the referral of patients with CMP by the GP. The referral factors were most often related to physical, psychological or GP factors. Important results were the apparent absence of social factors used for referral and the unfamiliarity of the GP with the treatment options. The factors mentioned by the participants should be taken into account when setting up a decision support tool for improving the referral process of patients with CMP.

## Supplementary Information

**Additional file 1.**

**Additional file 2.**

**Additional file 3.**

## Data Availability

The interview transcript generated during the current study are not publicly available, but data may be made available to interested parties from the corresponding author on reasonable request.

## Abbreviations

ADL: Activities of daily living
COVID: SARs-CoV-2 coronavirus
CMP: Chronic musculoskeletal pain
GP: General practitioner
SCEGS: Somatic, cognitive, emotional, behavior, social
