# Supplementary material for: Factors used by general practitioners for referring patients with chronic musculoskeletal pain: a qualitative study
Source: BMC Prim Care. 2022 May 24;23:126. doi: 10.1186/s12875-022-01743-6 (PMC9129062; doi:10.1186/s12875-022-01743-6)
Supplement: Supplementary file 1 — Additional file 1. [file 12875_2022_1743_MOESM1_ESM.docx]

# **Appendix 1. Semi-structured interview scheme**

**Demographic information**

- Age
- Sex
- Years working as a GP
- Working in a village or city
- Working in a solo- or group practice
- How would you rate your affinity with patients with CMP (0-10)?
- To what extent are you satisfied with your referral of patients with CMP (0-10)?

**General**
*This interview starts with some general questions about the referral of patients with CMP.*

1. When thinking about patients with CMP, do you distinguish between specific groups or characteristics of patients?
2. Based on which criteria do you refer a patient with CMP? (guidelines, (refresher) training)?
3. Which sources do you use for the referral of patients with CMP? And when you do deviate from these sources?
   1. Do you use questionnaires for referral? If yes, which?
   2. Do you use other measurement instrument for referral? If yes, which?
   3. Do you use observations for referral? If yes, which?
4. Which possible treatment options are there in the 1^st^, 2^nd^ and 3^rd^ line?

**1^st^ line**

*The next questions are about the referral of patients with CMP to specific healthcare providers.*

1. Which patient characteristics are associated with a referral to the 1^st^ line psychologist?
2. Which patient characteristics are associated with a referral to the psychosomatic physiotherapist?
3. Which patient characteristics are associated with a referral to the manual physiotherapist?
4. Which patient characteristics are associated with a referral to the regular physiotherapist or exercise therapist?
5. Which patient characteristics are associated with a referral to the lifestyle coach?
6. Which patient characteristics are associated with a referral to the practice nurse mental health?
7. Which patient characteristics are associated with a referral to the practice nurse for somatic complaints?
8. Which patient characteristics are associated with a referral to the 1^st^ line occupation therapist?
9. Which patient characteristics are associated with a referral to the 1^st^ line psychomotor therapist?
10. Which patient characteristics are associated with a referral to the 1^st^ line social worker?
11. Which patient characteristics are associated with a referral to an alternative therapist?
12. Which patient characteristics are associated with a referral to one-and-a-half-line care?

**2^nd^/3^rd^ line**

1. Which patient characteristics are associated with a referral to interdisciplinary pain rehabilitation?
2. Which patient characteristics are associated with a referral to the pain center?
3. Which patient characteristics are associated with a referral to the rehabilitation clinic?
4. Which patient characteristics are associated with a referral to the clinic for patients with medically unexplained physical symptoms?
5. Which patient characteristics are associated with a referral to the mental healthcare clinic?
6. Are there any other referral options and which patient characteristics are associated with a referral to this healthcare provider?

**Other/no referral**

1. What is your policy when a referred patient has to wait on a waiting list?
   1. What do you need when this is the case?
2. When do you not refer?
3. When do you choose to treat a patient with CMP with medication?
4. Which patients with CMP keep coming back to your consultation hours?
   1. How you deal with this?
5. When do you stop referring a treated patient that you think would not benefit from another treatment?
   1. Can you describe this patient?

**Patient factors**
*The next questions are about different patient related factors that could influence the referral of a patient with CMP.*

1. Which demands of the patient are considered when referring?
   1. Does the somatic orientation of the patient influence the referral?
   2. How does the patient, not accepting his/her pain as somatic inexplainable, influence the referral?
   3. How does the environment of the patient influence the referral?
   4. Does the patient’s insurance influence the referral? If yes, how?
   5. How does the occupational physician influence the referral?

**External factors**

1. Which factors related to different healthcare providers, other than the content of treatment, influence the referral of patients with CMP?

**GP factors**

1. Did you receive information about CMP during your GP education?
2. Did do follow refresher training about CMP?
   1. How long ago?
   2. Why did you (not) follow this training?
   3. What was the quality of this training?

**Final questions**

1. Are there any questions related to the referral of patients with CMP that we did not ask yet?
2. What should be changed in regard to the current referral process of patients with CMP?
   1. To what extent would a decision-support tool help?
   2. How do you think you would use this tool?
   3. When would you use this tool?

**Thank you**

*This were all the questions. Thank you very much for participating in this study and if you like we can keep you posted about the results.*
