# Supplementary material for: Factors used by general practitioners for referring patients with chronic musculoskeletal pain: a qualitative study
Source: BMC Prim Care. 2022 May 24;23:126. doi: 10.1186/s12875-022-01743-6 (PMC9129062; doi:10.1186/s12875-022-01743-6)
Supplement: Supplementary file 2 — Additional file 2. [file 12875_2022_1743_MOESM2_ESM.docx]

# **Appendix 2. All codes by treatment categories**

| Referral factors | Total | Description (if needed) |
| --- | --- | --- |
| Physical factors |  |  |
| 1. Somatic explainability | 10 |  |
| 1. Location pain complaints | 10 |  |
| 1. Specific clinical picture | 8 |  |
| 1. Type of pain complaint | 8 |  |
| 1. Duration pain complaint | 7 |  |
| 1. Function pain | 2 | The pain has a function for the patients |
| 1. Operable | 2 |  |
| 1. Pain intensity | 1 |  |
| 1. Outcome physical examination | 1 |  |
| Psychological factors |  |  |
| 1. Psychological complaints | 10 |  |
| 1. Level of acceptation treatment | 9 |  |
| 1. Level of acceptation pain | 6 |  |
| 1. DSM-5 diagnosis | 3 | Psychiatric diagnosis (from DSM-5 handbook) |
| 1. Stress | 2 |  |
| 1. Burn-out | 1 |  |
| Complaint factors |  |  |
| 1. Comorbidity | 8 |  |
| 1. Complexity | 8 |  |
| 1. Vicious cycle | 6 |  |
| 1. Tension | 4 |  |
| 1. Headache | 2 |  |
| 1. Tiredness | 2 |  |
| 1. Singular complaint | 2 | The patient only has 1 complaint |
| 1. Forgetfulness | 1 |  |
| 1. Concentration problems | 1 |  |
| 1. Problems with sleeping | 1 |  |
| 1. Urgency | 1 |  |
| 1. Occasion pain complaints | 1 | Dependent on the moment the patient has pain |
| 1. Level of suffering | 1 |  |
| 1. Fear of moving | 1 |  |
| 1. Psychosomatic complaints | 1 |  |
| Patient factors |  |  |
| 1. Treatment history | 10 |  |
| 1. Functioning | 10 |  |
| 1. Referral request patient | 10 |  |
| 1. Motivation patient | 7 |  |
| 1. Loadability | 6 |  |
| 1. Insurance | 6 |  |
| 1. Lifestyle | 5 |  |
| 1. Insight patient | 4 |  |
| 1. Request for help | 4 | Patient has a specific request |
| 1. Age patient | 4 |  |
| 1. Coping | 4 |  |
| 1. Caregiver | 3 | Patient is caregiver or not |
| 1. Intelligence patient | 3 |  |
| 1. Social economic status | 2 |  |
| 1. Personality traits | 2 |  |
| 1. Experience body | 2 | Patient knows their own body |
| 1. Social obligation | 2 |  |
| 1. Gender patient | 1 |  |
| General practitioner factors |  |  |
| 1. Knowlegde GP with treatment | 9 |  |
| 1. Professional opinion GP | 8 |  |
| 1. Competence GP | 7 |  |
| 1. Expected recovery period | 6 |  |
| 1. Experience GP | 6 |  |
| 1. Advice therapist or doctor | 6 |  |
| 1. NHG standard | 3 | Dependent on the GPs’ guidelines |
| 1. Referral options | 2 |  |
| 1. Doctor-patient relation | 1 |  |
| 1. SOLK standards | 1 |  |
| Treatment factors |  |  |
| 1. Experience with treatment | 9 |  |
| 1. Distance treatment | 7 |  |
| 1. Waiting list treatment | 6 |  |
| 1. Waitlist bridging | 6 |  |
| 1. Availability treatment | 3 |  |
| 1. Objective treatment | 1 | Dependent on the objective of a treatment |
| 1. Specialist referral needed | 1 |  |
| 1. Accessibility treatment | 1 |  |
| External factors |  |  |
| 1. Social environment | 4 |  |
| 1. Financial situation | 4 |  |
| 1. Problems at home | 4 |  |
| 1. Problems at work | 3 |  |
| 1. Influence environment patient | 3 |  |
| 1. Financial problems | 3 |  |
| 1. Problems in social life | 2 |  |
| 1. Work patient | 2 |  |
| 1. Work type | 2 |  |
| 1. Work physical activity | 2 | Dependent on the amount of physical activity the patient endures at work |
| 1. Problems social-cultural | 2 |  |
| 1. Situation at home | 2 |  |
| 1. Specialist referral | 2 |  |
| 1. Automatic referral | 1 |  |
| 1. Second opinion | 1 |  |
| 1. Course of medication use | 1 |  |
| 1. Personal injury claim | 1 |  |
