# Supplementary material for: Factors used by general practitioners for referring patients with chronic musculoskeletal pain: a qualitative study
Source: BMC Prim Care. 2022 May 24;23:126. doi: 10.1186/s12875-022-01743-6 (PMC9129062; doi:10.1186/s12875-022-01743-6)
Supplement: Supplementary file 3 — Additional file 3. [file 12875_2022_1743_MOESM3_ESM.docx]

**Appendix 3. Interviewguide focus group**

Introduction

Background information about the research, including: members of research group, problem statement and research goal.

Case

To better discuss referral factors for patients with CMP we started with a case, a patient of the rehabilitation physician:

Ms. X:

- Chronic low back pain since 2 years

- Pain in right buttock, with referring pain to anterior and right side of upper leg

- Pain is continuous and worsens during activity

- Sleep- and pain medication

- Tired and tensed up

Question: what else do you need to know about Ms. X before referring her for treatment?

Results general

The themes and codes as shown in this figure were explained per theme.


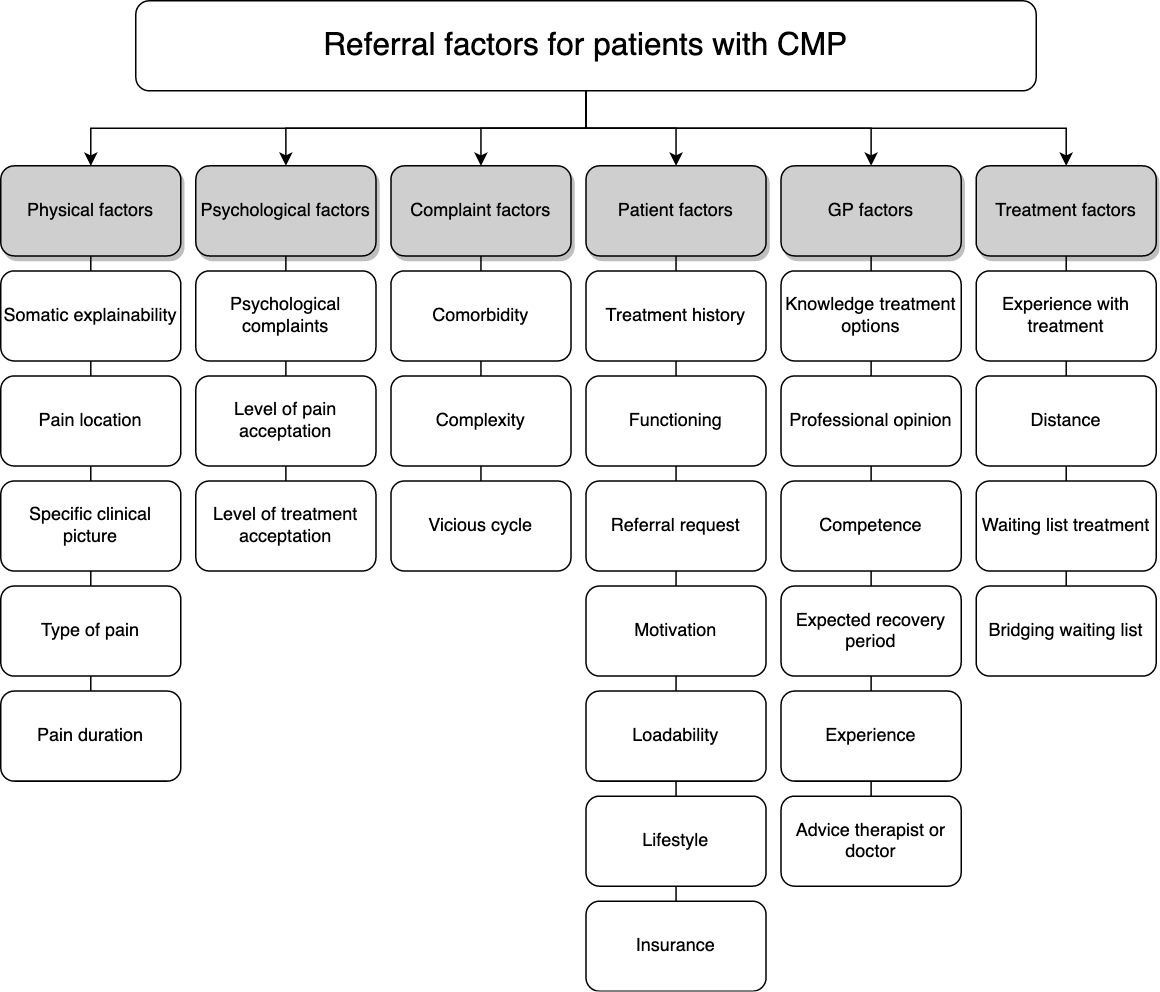


Questions:

1. What stands out for you?
2. Which themes and/or codes are missing?

Results specific codes

The most important codes per healthcare provider were explained and next the following questions were asked.

Questions:

1. What stands out for you?
2. Which themes and/or codes are missing?

Statements

The following two statements were discussed:

1. Social factors should be addressed more than they are now in the referral of patients with CMP.
2. The familiarity of local treatment options for patients with CMP should be increased for GPs.
